# Degarelix therapy for prostate cancer in a real-world setting: experience from the German IQUO (Association for Uro-Oncological Quality Assurance) Firmagon® registry

**DOI:** 10.1186/s12894-015-0116-4

**Published:** 2015-12-16

**Authors:** Götz Geiges, Thomas Harms, Gerald Rodemer, Ralf Eckert, Frank König, Rolf Eichenauer, Jörg Schroder

**Affiliations:** Arztpraxis für Urologie (Partnerpraxis der Charité), Berlin, Germany; Gemeinschaftspraxis Urologikum, Köln, Germany; Praxisgemeinschaft für Onkologie und Urologie, Wilhelmshaven, Germany; Urologische Arztpraxis, Lutherstadt Eisleben, Germany; ATURO – Praxis für Urologie, Berlin, Germany; Urologikum Hamburg, Hamburg, Germany

**Keywords:** Degarelix, Prostate cancer, Registry

## Abstract

**Background:**

We investigated the use of the gonadotropin-releasing hormone (GnRH) antagonist degarelix in everyday clinical practice using registry data from uro-oncology practices in Germany.

**Methods:**

Data were analysed retrospectively from the IQUO (Association for uro-oncological quality assurance) patient registry. Data were prospectively collected from all consecutive PCa patients treated with degarelix (*n* = 1010) in 138 uro-oncology practices in Germany between May 2009 and December 2013.

**Results:**

Median overall survival had not yet been reached in the all-patient group or in subgroups who had or had not received prior hormonal therapy (HT). Cox regression analysis showed that patients who had received prior HT (*n* = 542) had a 58 % increased mortality risk (hazard ratio 1.58, 95 % CI 1.20–2.09) versus patients who had not (*n* = 468) (*p* = 0.001). Also, in patients who had received prior luteinizing hormone-releasing hormone (LHRH) analogue therapy (LHRH agonists or GnRH antagonists), median time to PSA progression was shorter (209 weeks) than in those who had not received prior LHRH analogues (*n* = 555; median PSA progression-free survival not yet reached). Degarelix was generally well tolerated.

**Conclusions:**

Degarelix was effective and well tolerated in everyday clinical practice, confirming observations from clinical studies. Patients who received prior HT appeared to have a significantly higher mortality risk.

## Background

The gonadotropin-releasing hormone (GnRH) antagonist, degarelix, is an effective and well tolerated treatment for advanced prostate cancer (PCa) [1–3]. As an antagonist, degarelix benefits from a direct mechanism of action that inhibits GnRH without causing an initial surge in gonadotropins or consequently testosterone [4]. Degarelix displays similar efficacy to the luteinizing hormone-releasing hormone (LHRH) agonist, leuprolide, for testosterone suppression in PCa [1]. However, testosterone reduction was more rapid with degarelix and, unlike leuprolide, occurred without a testosterone surge or microsurges; in advanced disease, the testosterone surge associated with LHRH agonists can produce symptom flare [5]. Degarelix also displayed superior prostate-specific antigen (PSA) progression-free survival (PFS) compared with leuprolide [6] and in metastatic disease was associated with better control of the bone formation marker serum alkaline phosphatase (S-ALP), suggesting that it might offer prolonged control of skeletal metastases [7].

While degarelix has been well studied in clinical trials, it is important to examine its efficacy and tolerability in everyday clinical practice. Clinical trials give important data about treatment effects in controlled conditions, but patient registries offer additional data from a wide population (with few excluded patients), allowing evaluation of care as actually provided [8]. Consequently, registry data are likely to provide a more representative indication of the real-world patient experience with degarelix. Therefore, the current study investigated the use of degarelix in patients from uro-oncology practices in Germany by analysing data from the IQUO (Association for uro-oncological quality assurance) Firmagon® patient registry.

## Methods

Data were analysed retrospectively from the IQUO (Association for uro-oncological quality assurance) patient registry records using the QuaSi-URO® documentation system, a live system based on data from IQUO members. Data from the Firmagon® Registry (The Electronic Therapy Documentation [ETD] Firmagon® – Urology) were prospectively collected from all consecutive patients with PCa treated with degarelix in 138 uro-oncology practices in Germany between 27 May 2009 and 10 December 2013. All patients with PCa receiving degarelix who were entered into ETD Firmagon® were documented and all patients with at least one fully documented entry in the registry were included. The only criterium for inclusion into the registry was prescription of degarelix, thus everyday usage should be mirrored by this register. The decision to treat patients with degarelix was taken by the treating physician before the decision for inclusion into the registry.

The underlying data collection for this publication was performed in accordance with the principles of the Declaration of Helsinki. The data collection did not take place in a study environment but utilized electronic data capture according to the documentation system and documentation guidelines of the IQUO. Within the electronic data capture there is no personal or patient identifying information; data were collected anonymously and pseudonymised. Identification of patients is possible only by the physician and the documentation staff. Therefore, according to three independent legal opinions there was no need for ethics committee approval or a patient consent form.

### Study variables

The data repository was reviewed for information on the following variables: tumour response to degarelix; overall survival (OS); PSA PFS; percentage change in median PSA and testosterone over time; percentage of patients with PSA ≤ 4 ng/ml over time; percentage change in median S-ALP over time; change in mean prostate volume. These variables were measured for patient subgroups as summarized in Table 1. Prior hormonal therapy (HT) included LHRH agonists, GnRH antagonists or antiandrogens. Prior LHRH analogue therapy included LHRH agonists or GnRH antagonists. Recording of the testosterone, S-ALP and prostate volume at the time of documentation was optional.

**Table 1:** Summary of efficacy evaluations according to patient subgroup

	Patient subgroup						
Evaluation	All patients	Patients with metastatic disease	Prior HT^a^	No prior HT	Baseline PSA ≥20 ng/ml	Prior LHRH analogue therapy^b^	No prior LHRH analogue therapy
Tumour response	√						
Overall survival	√		√	√		√	√
PSA progression-free survival	√	√				√	√
Percentage change in median PSA	√	√	√	√			
Percentage change in median testosterone	√	√	√	√			
Percentage change in median S-ALP		√		√			
Mean prostate volume	√						
Percentage of patients with PSA ≤ 4 ng/ml	√	√			√		

^a^LHRH agonists, GnRH antagonists or antiandrogens; ^b^LHRH agonists or GnRH antagonists

Tumour response (objective progression or stabilization, complete or partial remission) was assessed using the Response Evaluation Criteria In Solid Tumors [RECIST] guidelines [9] and measured at 6-month intervals up to a total duration of 3 years. Median time to PSA progression was defined according to Prostate Cancer Clinical Trials Working Group [PCWG2] criteria: a ≥25 % increase and an absolute increase of ≥2 ng/ml from the nadir, confirmed by a second value ≥3 weeks later [10]. At the time of PSA progression, patients were also required to have testosterone levels < 0.5 ng/ml [castration level]. The date of PSA progression was the date of the first PSA test value.

Percentage change in median PSA, testosterone and S-ALP, and mean prostate volume were measured monthly during the first year of treatment and thereafter in 3-monthly intervals for up to 39 months. Percentage change in median PSA and percentage of patients with PSA ≤ 4 ng/ml are presented for 3-month intervals for up to 36 and 24 months, respectively. Longer term data is not shown due to the smaller patient numbers towards the end of the evaluation period, especially in subgroups. Since collection of testosterone, ALP and prostate volume data was optional, patient numbers are smaller and therefore percentage change in median testosterone is presented for up to 2 years (1 year in metastatic disease subgroup), S-ALP for up to 1 year and mean prostate volume for up to 6 months.

The first data point in the registry refers to the time of the first administration of degarelix (baseline). In ongoing treatments, the last data point refers to the time of the last documented administration of a degarelix dose.

### Statistics

Change in PSA, S-ALP, and prostate volume are summarized using descriptive statistics (percentage change, percentage change in median, mean). OS and PSA PFS were analysed using the Kaplan–Meier method. Survival was calculated from the date of first treatment until the date of any cause of death.

## Results

### Patients

A total of 1,010 patients received degarelix and were included. Baseline demographic and disease characteristics of the registry population are summarized in Table 2. Around two-thirds of patients had localized (*n* = 469) or locally advanced (*n* = 201) disease (340 had metastatic disease) and almost half had a Gleason score of 8–10. A total of 542 patients had received prior HT, predominantly LHRH agonists (53.7 %); of these, 455 had received prior LHRH analogue therapy (LHRH agonists or GnRH antagonists). Baseline PSA (median 14.27 ng/ml) was ≥20 ng/ml in 43.6 % of patients.

**Table 2:** Baseline patient characteristics

Characteristic	*N* = 1,010 (100)
Mean (SD) age,^1^ years	70.29 (8.55)
Mean (SD) BMI,^1^ kg/m^2^	26.88 (3.81)
Median (range) PSA ng/ml	14.27 (0–6131)
Median (range) testosterone ng/ml (*n* = 266)^a^	0.52 (0–34.45)
Testosterone categories n, % (*n* = 266)	
<0.1 ng/ml	26 (9.8)
0.1– ≤ 0.2 ng/ml	54 (20.3)
0.2–0.5 ng/ml	48 (18.1)
> 0.5 ng/ml	138 (51.9)
Disease stage n (%)	
Localized^b^	469 (46)
Locally advanced^c^	201 (20)
Metastatic^d^	340 (34)
Gleason score n, %	*N* = 868
2–4	13 (1.5)
5–6	130 (15.0)
7	290 (33.4)
8–10	416 (47.9)
Not classified	19 (2.2)
PSA categories n, %	
< 10 ng/ml	438 (43.4)
10– ≤ 20 ng/ml	133 (13.1)
20–50 ng/ml	160 (15.8)
> 50 ng/ml	279 (27.6)
Previous treatment	
Watchful waiting (%)	
N	829 (82.1)
Y	181 (17.9)
External radiotherapy (%)	
N	783 (77.5)
Y	227 (22.5)
Brachytherapy (%)	
N	990 (98.0)
Y	20 (2.0)
High-intensity focused ultrasound(%)	
N	1002 (99.2)
Y	8 (0.8)
Radical prostatectomy (%)	
N	764 (75.6)
Y	246 (24.4)
Hormone therapy (%)	
N	468 (46.3)
Y	542 (53.7)
LHRH agonists	309 (57.0)
GnRH antagonist	146 (26.9)
Antiandrogen	87 (16.1)
Palliative therapy (%)	
N	766 (75.8)
Y	244 (24.2)

Baseline is defined as the beginning of degarelix therapy. ^a^Documentation of the testosterone value was optional; ^b^Localized disease: T1/T2, NX or N0, and MX or M0; ^c^Locally advanced disease: T3/T4, NX or N0, and MX or M0; ^d^Metastatic disease: N1 or M1, any T

^1^Recorded at the time of initial diagnosis of PCa

At the time of this report, a total of 401, 158 and 44 patients had received degarelix for 12, 24 and 36 months, respectively; only two patients had received treatment for 48 months, reflecting entry to the register over time, at the time of this analysis.

Reasons for discontinuation of degarelix in patients with documented answers (*n* = 479) included change in therapy (30.7 %), patient request/others (22.8 %), death (14.2 %), PSA progression (14.4 %), clinical progression (7.5 %), adverse event (6.1 %) or lost to follow-up (4.4 %).

### Tumour response

**Fig. 1:**
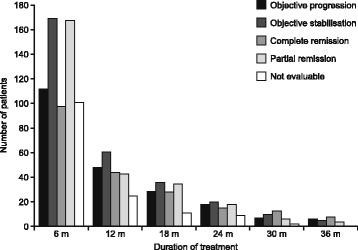
Patient response to treatment over time

After 1 year of treatment, among patients with a documented response (*n* = 221), 19.9 % (*n* = 44) had complete remission, 19.5 % (*n* = 43) partial remission, 27.6 % (*n* = 61) objective stabilization and 21.7 % (*n* = 48) objective progression; 11.3 % (*n* = 25) were not evaluable. After 24 months (*n* = 80), these values were 18.8 % (*n* = 15), 22.5 % (*n* = 18), 25.0 % (*n* = 20), 22.5 % (*n* = 18) and 11.3 % (*n* = 9), respectively. After 36 months, (*n* = 23), these values were 34.8 % (*n* = 8), 17.4 % (*n* = 4), 21.7 % (*n* = 5), 26.1 % (*n* = 6) and 0 % (*n* = 0), respectively. Patient responses over time are summarized in Fig. 1*.*

### Overall survival

In total, 212 of 1,010 patients died and 51 were lost to follow-up. The median OS (all patients) had not yet been reached; the 75th percentile value (i.e. where 25 % of patients had reached event) was 148.9 weeks. While the median OS had also not yet been reached in patients with or without prior HT, the 75th percentile value was longer for those without prior HT (176.4 weeks) compared with those with prior HT (131.7 weeks). A Cox regression analysis showed patients who received prior HT (*n* = 542) had a 58 % increased mortality risk (hazard ratio 1.58, 95 % CI 1.20–2.09) compared with patients who had not received prior HT (*n* = 468) (*p* = 0.001).

Similarly, median OS had not been reached in patients with or without prior LHRH analogue therapy (LHRH agonist/GnRH antagonist), however, the 75th percentile value was shorter (123.1 weeks) in those who received prior LHRH analogues compared with those who had not (167.4 weeks).

### PSA PFS

Of the 1,010 patients in this study, 200 (20 %) experienced PSA progression, 137 patients died, 41 were lost to follow-up and 632 were alive and PSA progression-free. Median PSA PFS was not yet reached; the 75th percentile value for PSA PFS was 59.6 weeks.

Of the 340 patients with metastatic disease, 91 (27 %) experienced PSA progression. Median time to PSA progression (PSA PFS) was 141.4 weeks (75th percentile value 32.4 weeks).

In patients who received prior LHRH analogue therapy (*n* = 455), median time to PSA progression was shorter (209 weeks [75th percentile value 30.4 weeks]) than in those who had not received prior LHRH analogues (*n* = 555) where median PSA PFS was not reached (75th percentile value 88.4 weeks). Also, a greater proportion of patients who received prior LHRH analogue therapy (25.05 %; *n* = 114) experienced PSA progression compared with those who had not (15.5 %, *n* = 86).

### PSA

**Fig. 2:**
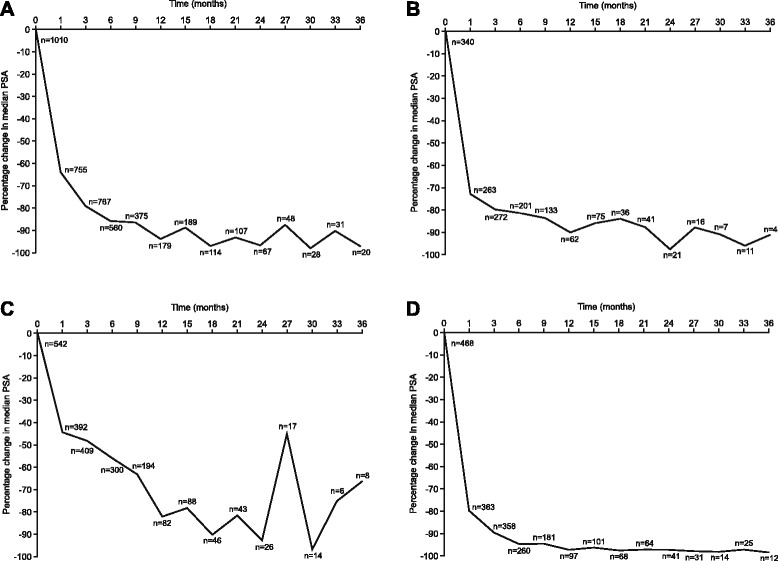
Percentage change in median PSA over time in (**a**) all patients; **b** patients with metastatic disease at baseline; **c** patients with prior hormonal therapy; and (**d**) patients with no prior hormonal therapy

Overall, degarelix produced a rapid and profound PSA reduction, sustained for up to 36 months (Fig. 2a). A similar PSA profile was observed in patients with baseline metastatic disease (Fig. 2b). PSA suppression was greater and more rapid, and maintenance of PSA suppression more effective, in patients who had not received prior HT compared with those who had received prior HT (Fig. 2c–d).

**Fig. 3:**
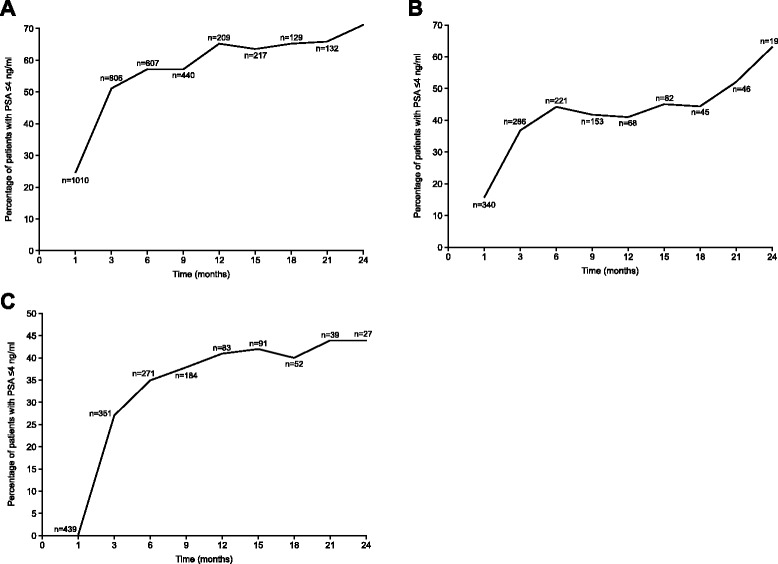
Percentage of patients with PSA ≤ 4 ng/ml over time for (**a**) all patients; **b** patients with metastatic disease at baseline; and (**c**) patients with baseline PSA ≥20 ng/ml

Overall, PSA reduction to ≤ 4 ng/ml was achieved in 65 % of patients at 12 months and 71 % of patients at 24 months (Fig. 3a). These values were lower in patients with metastatic disease (41 % and 63 %, respectively) and patients with baseline PSA ≥20 ng/ml (41 % and 44 %, respectively); (Fig. 3b–c).

### Testosterone

Overall, degarelix produced a rapid and profound reduction in testosterone (an optional measurement), falling to 0.19 ng/mL after 1 month (*n* = 217). Testosterone suppression <20 ng/mL was sustained for the first 24 months in the all-patient group (median testosterone 0.13 ng/mL at 24 months [*n* = 18]). Testosterone was also suppressed in patients with baseline metastatic disease: median testosterone level was 0.2 ng/mL after 1 month (*n* = 92) and 0.13 ng/mL at 12 months (*n* = 20).

### S-ALP

**Fig. 4:**
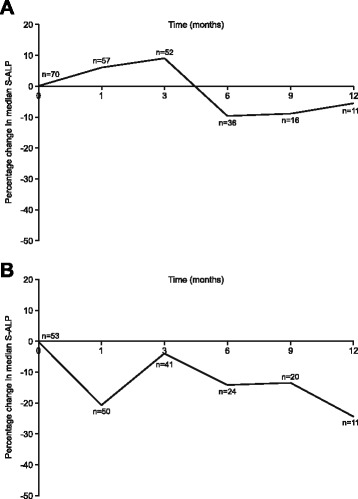
Percentage change in median serum alkaline phosphatase over time in (**a**) patients with metastatic disease at baseline and (**b**) patients with no prior hormonal therapy

Changes in S-ALP (an optional measurement) over time for patients with metastatic disease (*n* = 70 at 0 months and *n* = 11 at 12 months) and those without prior HT (*n* = 53 at 0 months and *n* = 11 at 12 months) are summarized in Fig. 4. In the metastatic cohort, S-ALP was suppressed after 6–12 months of treatment. S-ALP suppression was observed after 1 month in patients with no prior HT and this was maintained for up to 12 months.

### Prostate volume

Mean prostate volume decreased from 36.83 ml at baseline (*n* = 85 patients) to 30.32 ml (*n* = 25) after 3 months and to 26.38 ml after 6 months (*n* = 16).

### Tolerability

The most frequent adverse events recorded in patients included hot flushes (12.9 %), injection site erythema (8.5 %), fatigue (5.2 %) and pain (4.2 %). Adverse events experienced by patients at the first and last data points during degarelix therapy are summarized in Table 3. Apart from a slight increase in the incidence of hot flushes, there were no significant changes in frequency of adverse events between the first and last data points.

**Table 3:** Adverse events at first and last data points during degeralix therapy

Adverse event n,%	First data point^a^	Last data point^b^
Total^c^	276 (27.3)	380 (37.6)
Hot flushes	80 (7.9)	130 (12.9)
Erythema at the injection site	61 (6.0)	86 (8.5)
Fatigue	41 (4.1)	53 (5.2)
Pain	29 (2.9)	42 (4.2)
Weight gain	20 (2.0)	20 (2.0)
Back pain	10 (1.0)	18 (1.8)
Hypertension	12 (1.2)	6 (0.59)
Cardiac arrhythmia	3 (0.3)	3 (0.3)
Thromboembolism	0	0
Heart attack	2 (0.2)	1 (0.1)
Arteriosclerosis	0	1 (0.1)
Osteoporosis	0	1 (0.1)
Others	18 (1.8)	19 (1.9)

^a^At the time of the first degarelix dose; ^b^at the final degarelix dose; ^c^percentages are stated as a percentage of the total patient population

## Discussion

Patient registries offer a unique and powerful tool for the collection of observational and clinical data, helping provide a clearer understanding of a therapy's impact on patients in a real-world context. While patient populations in clinical trials are tightly controlled, with rigorous selection criteria, registries generally have much broader inclusion criteria and fewer exclusion criteria which can lead to greater generalizability [11].

Degarelix is a GnRH antagonist for the first-line treatment of androgen-dependent advanced PCa. However, comparison of patient populations in the Firmagon registry versus clinical trials shows that while degarelix trials excluded patients with previous hormonal management of PCa (except those who underwent localized therapy of curative intent in which neoadjuvant or adjuvant HT for ≤ 6 months was accepted) [1, 3, 12, 13], over half the patients in the current registry had received prior HT (30 % of the total population had received prior LHRH agonists). It was observed that, in this study of real-world experience with degarelix therapy, of those with documented answers, degarelix was discontinued in 30.7 % of patients due to a change in therapy and in 22.8 % as a result of patient request/other reasons compared with 14.4 % of patients discontinuing due to PSA progression and 7.5 % due to clinical progression. Information regarding the reasons for patients changing therapy was not collected in the current analysis; reasons for changing treatment may possibly include higher expectations regarding efficacy and/or lowering side effects.

Also, 34 % of the registry population had baseline metastastic disease compared with around 20 % [1, 12, 13] to 23 % [3] in clinical trials. Interestingly, in our registry, 46 % of patients who received degarelix had localised disease and over half had PSA ≤20 ng/mL. In the community setting, ADT has been used commonly as primary therapy for localised prostate cancer [14] particularly in the elderly [15]. However, European Association of Urology guidelines consider androgen suppression to be unsuitable as primary therapy for low-risk prostate cancer and consider that, in patients with non-metastatic localised disease not suitable for curative treatment, immediate ADT should be used only in patients requiring symptom palliation [16].

In the current study, degarelix achieved a complete response in 20 % of patients after 1 year and 19 % after 2 years; although this value rose to 35 % at 3 years, patient numbers at this time were small. Partial responses were noted in 20 %, 22.5 % and 17 % of patients, after 1, 2 and 3 years, respectively. In the degarelix trial of Ozono et al. [3], the best overall response (complete response [3.6 %] + partial response [67.9 %]) was 71.4 % in the 240/80 mg group.

In our registry study, patients with prior HT appeared to have a significantly higher mortality risk (hazard ratio 1.58) compared with those who had not received prior HT. Castration resistance and concomitant progression is inevitable in these patients and it is not surprising that prior HT shortens the observed remission time for every other androgen deprivation therapy, such as degarelix, that commences thereafter*.* Also, in the subgroup who had received prior LHRH analogues (LHRH agonist/GnRH antagonist), the 75th percentile value for OS was shorter than in those not pre-treated with LHRH analogues. Similarly, a higher percentage of patients who had received prior LHRH analogues experienced PSA progression compared with those who had not, and median time to PSA progression was shorter (209 weeks) in the subgroup who had received prior LHRH analogues versus those who had not (median PSA PFS not reached). These results appear to compare favourably with earlier studies of ADT. One large study of ADT in previously untreated PCa patients showed a median PFS of 16.5 months and 13.9 months, with LHRH agonists with and without antiandrogen, respectively [17]. In other studies in previously hormone-naïve patients, similar times to progression have been noted with LHRH agonists with/without antiandrogens (in the range of 12 to 23 months) [18–22].

Data from clinical studies show that degarelix displayed superior PSA PFS compared with leuprolide over 1 year [6], and there was an improvement in PSA PFS in patients who crossed over from leuprolide to degarelix in a long-term extension trial [2]. Furthermore, a pooled analysis also showed improved PSA PFS and longer OS (likely due to a decreased risk of cardiovascular disease) with degarelix compared with LHRH agonists [23]. Indeed, compared to LHRH agonists, GnRH antagonists appear to halve the number of cardiac events experienced by men with pre-existing cardiovascular disease during the first year of ADT [24]. Improvements in PSA PFS are indicative of delayed progression to castration-resistant disease with degarelix. Interestingly, since over half of the registry patients had received prior HT, it was not expected to achieve the same efficacy as in controlled studies which generally excluded patients who had prior HT. Nevertheless, the current registry data indicate that degarelix provides some benefit in patients who were pre-treated with HT (e.g. LHRH-agonists), while most benefit was observed in hormone-naïve patients.

PSA suppression in registry patients with no prior HT was more rapid and effective than those pre-treated with HT; moreover, the hormone-naïve cohort showed a PSA suppression profile similar to that observed with degarelix in clinical trials where previous hormonal management of PCa was excluded.

In the registry patients, PSA reduction to ≤ 4 ng/ml was achieved in 65 % of patients at 12 months and 71 % at 24 months. In the pivotal phase III degarelix clinical trial (CS21), the proportion of patients achieving PSA suppression < 4 ng/ml was 83 % after 1 year [6]. The difference most likely reflects the fact that patients in the registry had a higher risk compared to the registration trial, CS21. Over time, the proportion of registry patients with metastatic disease who achieved PSA suppression < 4 ng/ml was lower than the overall registry population; similarly, the proportion of patients with metastatic disease in the CS21 trial achieving PSA suppression < 4 ng/ml was also lower than the overall study population. Southwest Oncology Group trial S9346 data showed that PSA ≤ 4 ng/ml after ADT is a strong predictor of survival [25]. After controlling for prognostic factors, patients with PSA ≤ 4 to > 0.2 ng/ml had less than one-third the risk of death versus those with PSA > 4 ng/ml; median survival was 13 months for patients with PSA > 4 ng/ml versus 44 months for patients with PSA > 0.2 to ≤ 4 ng/ml.

The registry data also showed that overall, degarelix produced a rapid and profound testosterone suppression that was sustained for up to 24 months. Testosterone was also suppressed in patients with baseline metastatic disease. Testosterone measurement was optional and so, over time, patient numbers were small.

S-ALP is a marker of bone formation and baseline levels are high in metastatic disease, indicative of skeletal metastases [7]. Therefore, we examined the effect of degarelix in the cohort of patients with metastatic disease and found that S-ALP was suppressed after 6–12 months with degarelix. This compares with S-ALP suppression below baseline levels in the metastatic cohort after only 2 months of degarelix therapy in the CS21 trial [7]. A decrease in bone turnover marker levels may delay progression of bone metastases and improve survival.

Neo-adjuvant ADT can reduce prostate volume before radiotherapy. The registry data showed a decrease in prostate volume of almost 18 % at 3 months (and ~28 % at 6 months). This is slightly below the reported reductions (37–42 %) in prostate volume achieved with degarelix in clinical studies [26, 27]. As well as facilitating more effective delivery of radiotherapy, rapid and pronounced reduction of total prostate volume may also provide additional benefit for patients with obstructive lower urinary tract symptoms.

Degarelix was well tolerated in registry patients, with similar adverse event profiles for patients at the first and last data points. Moreover, the adverse event profile (based on adverse events at the last data point) was as expected for this patient population and similar to that of patients receiving degarelix in the 1-year phase II and III clinical trials, with the most frequent adverse events typically comprising injection-site reactions, hot flushes, and fatigue [1, 12, 13].

Some limitations of registry-based cohort studies may include limited availability of treatment data and underreporting of outcomes if a patient leaves the registry or is not adequately followed up [11]. In the current registry, over time, patient numbers for some parameters (especially e.g. testosterone, prostate volume, and S-ALP where recording was optional) became quite small in some patient subgroups (e.g. metastatic patients) which may affect the reliability of measurements towards the end of the observation period in these cases. Missing data are a common phenomenon in healthcare research projects; not all data provided are complete due to the non-interventional nature of this study type. The uncertainty based on this will be relativized by the collection and generation of more data. Where there is a significant quantity of missing data, this may bias or impact on the study finding. One reason for the reduced number of patients is that some patients at the time of the analysis had not yet reached the full follow-up period.

## Conclusion

This analysis of registry data showed degarelix to be effective and well tolerated in this real-world setting, confirming observations from controlled clinical studies. PSA-PFS appeared to be more favourable in comparison to earlier studies with other ADT treatments. Patients who had received prior HT appeared to have a significantly higher mortality risk than those who had not received prior HT.

## Abbreviations

ADT: androgen deprivation therapy
GnRH: gonadotropin-releasing hormone
HT: hormonal therapy
IQUO: association for uro-oncological quality assurance
LHRH: luteinizing hormone-releasing hormone
OS: overall survival
PCa: prostate cancer
PCWG: Prostate Cancer Clinical Trials Working Group
PFS: progression-free survival
PSA: prostate-specific antigen
RECIST: Response Evaluation Criteria In Solid Tumors
S-ALP: serum alkaline phosphatase
